# Operation for Imperforate Vagina

**Published:** 1841-08

**Authors:** John Travis

**Affiliations:** Carroll county, Tenn.


Art. VI.—*Operation for Imperforate Vagina.* By John Travis, M. D., of Carroll county, Tenn.

On the 13th day of May I was called to visit Miss Smith, a young lady of this county, aged 16 years. I found her laboring under severe pain of the hips, a sense of heaviness and tension in the uterus, and hardness of the abdomen. On enquiry of her parents, I learned that she had never menstruated, and that she had been under medical treatment for about two years, generally taking emmenagogue medicines. This treatment, instead of affording any relief, only increased the intensity of the pain. By consent of her parents, I made an examination per vaginam, and ascertained that there was no opening for the escape of the menstrual fluid.

I then performed the operation according to the plan of Cooper, by making an incision, more than an inch in length, just below the *meatus urinarius* downward. There was an immediate escape of three pints of *black blood.* The girl was at once relieved of pain, and in a few minutes left her bed and said she was well, and has continued to attend to her ordinary business without taking her bed.

There is danger in delaying this operation. When the blood accumulates in such quantities that the vagina cannot contain it, the uterus becomes distended, and the fluid may escape through the Fallopian tubes and be extravasated in the abdomen.

There can be no question that this operation will prolong the life of the patient on whom it was performed. The hymen was unusually thick and strong, so much so, that the malformation could not have been removed by the *vis medicatrix naturæ.*

*June* 3, 1841.

